# Rapamycin‐mediated mouse lifespan extension: Late‐life dosage regimes with sex‐specific effects

**DOI:** 10.1111/acel.13269

**Published:** 2020-11-04

**Authors:** Randy Strong, Richard A. Miller, Molly Bogue, Elizabeth Fernandez, Martin A. Javors, Sergiy Libert, Paul Anthony Marinez, Michael P. Murphy, Nicolas Musi, James F. Nelson, Michael Petrascheck, Peter Reifsnyder, Arlan Richardson, Adam B. Salmon, Francesca Macchiarini, David E. Harrison

**Affiliations:** ^1^ Geriatric Research, Education and Clinical Center and Research Service South Texas Veterans Health Care System San Antonio TX USA; ^2^ Department of Pharmacology Barshop Institute for Longevity and Aging Studies at The University of Texas Health Science Center at San Antonio San Antonio TX USA; ^3^ Department of Pathology and Geriatrics Center University of Michigan Ann Arbor MI USA; ^4^ The Jackson Laboratory Bar Harbor ME USA; ^5^ Department of Psychiatry University of Texas Health Science Center at San Antonio San Antonio TX USA; ^6^ Calico Labs South San Francisco CA USA; ^7^ Medical Research Council Mitochondrial Biology Unit University of Cambridge Cambridge UK; ^8^ Geriatric Research, Education and Clinical Center South Texas Veterans Health Care System San Antonio TX USA; ^9^ Department of Medicine Barshop Institute for Longevity and Aging Studies at The University of Texas Health Science Center at San Antonio San Antonio TX USA; ^10^ Department of Cellular and Integrative Physiology Barshop Institute for Longevity and Aging Studies at The University of Texas Health Science Center at San Antonio San Antonio TX USA; ^11^ Department of Molecular Medicine The Scripps Research Institute La Jolla CA USA; ^12^ Department of Neuroscience The Scripps Research Institute La Jolla CA USA; ^13^ Department of Biochemistry & Molecular Biology University of Oklahoma Health Science Center Oklahoma City OK USA; ^14^ Oklahoma City VA Medical Center Oklahoma City OK USA; ^15^ Department of Molecular Medicine Barshop Institute for Longevity and Aging Studies at The University of Texas Health Science Center at San Antonio San Antonio TX USA; ^16^ Division of Aging Biology National Institute on Aging Bethesda MD USA

**Keywords:** 17‐DMAG, minocycline, MitoQ, rapamycin, survival, β‐GPA

## Abstract

To see if variations in timing of rapamycin (Rapa), administered to middle aged mice starting at 20 months, would lead to different survival outcomes, we compared three dosing regimens. Initiation of Rapa at 42 ppm increased survival significantly in both male and female mice. Exposure to Rapa for a 3‐month period led to significant longevity benefit in males only. Protocols in which each month of Rapa treatment was followed by a month without Rapa exposure were also effective in both sexes, though this approach was less effective than continuous exposure in female mice. Interpretation of these results is made more complicated by unanticipated variation in patterns of weight gain, prior to the initiation of the Rapa treatment, presumably due to the use of drug‐free food from two different suppliers. The experimental design included tests of four other drugs, minocycline, β‐guanidinopropionic acid, MitoQ, and 17‐dimethylaminoethylamino‐17‐demethoxygeldanamycin (17‐DMAG), but none of these led to a change in survival in either sex.

## Graphical abstract

Intermittent (1 month on/1 month off) or a limited 3‐month exposure to rapamycin, beginning at 20 months, was as effective as continuous exposure in increasing survival in males. In females, only intermittent exposure increased survival but the increase was not as great as continuous exposure.

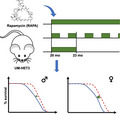

## 1 INTRODUCTION

The NIA Interventions Testing Program (ITP) showed in 2009 that male or female mice given the mTORC1 inhibitor rapamycin (Rapa) from age 20 months had a significant increase in median lifespan (Harrison et al., 2009) and also increased the proportion of mice alive at the 90th percentile survival age. Subsequent reports documented a similar degree of lifespan extension in mice given this drug starting from 9 months of age (Miller et al., 2011) and that the degree of extension was dependent on Rapa dose over the range of 4.7–42 parts per million (ppm) in food (Miller et al., 2014). The combination of Rapa with metformin led to as much as a 26% increase in female median lifespan and 23% increase in median male lifespan (Strong et al., 2016), although these survival results were not significantly higher than those noted in earlier experiments using the same dose (14.7 ppm) of Rapa by itself. Mice treated with Rapa (14.7 ppm) from 9 months and then euthanized at 22 months showed lower incidence of pathological changes in heart, tendon, adrenal, endometrium, and liver (Wilkinson et al., 2012), among other organs, suggesting that many aspects of aging were delayed or decelerated by Rapa, and that this retardation of age‐related changes was responsible for postponement of lethal illnesses and lifespan extension. Studies with higher doses of rapamycin, delivered either orally or intraperitoneally, also had beneficial effects on lifespan extension and on cancer incidence (Bitto et al., 2016).

Because it is possible that Rapa could create a mixture of beneficial and harmful effects, we considered the possibility that variations of dose timing might lead to further increases in lifespan when compared to continuous Rapa administration. We report here longevity results using three dosing schemes: “Rapa 20 mon” in which Rapa is given at 42 ppm from 20 months until death; “Rapa cycles” in which Rapa is given for 1‐month interval interspersed with drug‐free months from 20 months until death; and “Rapa 20–23,” in which Rapa is started at 20 months but then terminated at 23 months.

The ITP reports the outcome of each longevity experiment even when the agent tested did not lead to a convincing longevity benefit. In 2015, the cohort used for the Rapa dosing study (C2015), the ITP also tested four other agents: 17‐dimethylaminoethylamino‐17‐demethoxygeldanamycin hydrochloride (17‐DMAG), which inhibits HSP90 (Jez et al., 2003); Minocycline (Min), a member of a class of that includes tetracycline and doxycycline (Klein & Cunha, 1995); β‐GPA (β‐guanadinopropionic acid) (β‐GPA), which lowers both blood glucose and blood insulin levels, similar to the effects of dietary restriction (Ohira et al., 1994); and MitoQ, a small molecule antioxidant that accumulates in mitochondria in cultured cells and in vivo (Kelso et al., 2001; Smith et al., 2003; Smith & Murphy, 2010). None of these four agents led to a significant increase in lifespan.

## 2 RESULTS

**FIGURE 1:**
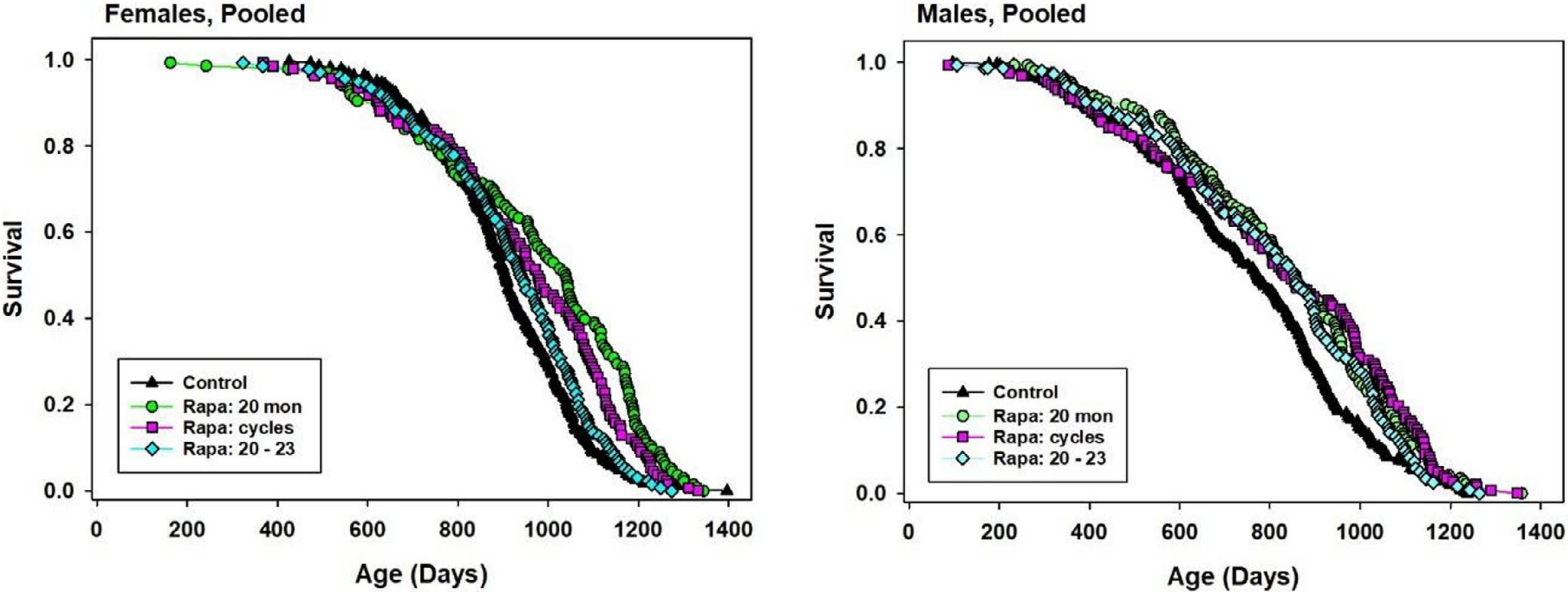
Kaplan–Meier survival plots for female and male mice exposed to different Rapa treatment schedules. Green circles: Rapa given from 22 months until death. Red squares: Rapa given from 20–21, 22–23, 24–25, etc., until death. Cyan diamonds: Rapa given for 3 months only, starting at 20 months. Data are pooled from all three test sites

Genetically heterogeneous male and female mice, from the four‐way UM‐HET3 cross, were given food containing Rapa at 42 ppm from 20 months of age at each of the three test sites. One group of cages (“Rapa 20”) received this dose for the rest of their lives. A second group (“Rapa cycles”) received Rapa for 1‐month period interrupted by 1 month periods without drug. The third group (“Rapa 20–23”) received Rapa for the 3‐month period starting at age 20 months, but not thereafter. Kaplan–Meier plots are shown in Figure 1, and summary statistics, pooled across sites, are collected in Table 1. In male mice, all three dosage schedules led to a similar increase in median lifespan (9%–11%), and all three produced a significant result by the log‐rank test. The Wang–Allison (WA) test, which we use as an index of extreme longevity, was significant for Rapa 20 and Rapa cycles, but not (*p* = 0.08) for the Rapa 20–23 protocol. Formal comparisons, using the log‐rank test, between each pair of treatment protocols found no significant difference among them for male mice (0.28 < *p* < 0.61).

**TABLE 1:** Survival statistics for mice treated with Rapamycin using various dosage schedules, pooled across sites

Rx	Group	Sex	Count	Median (days)	Percent change in median	Log‐rank *p*‐value	90th %ile (days)	Percent change in 90th %ile	WA *p*‐value
Controls	Pool	M	300	772			1049		
Rapa 20 mon	Pool	M	156	856	11	0.0007	1148	9	0.04
Rapa cycles	Pool	M	159	841	9	0.002	1144	9	0.001
Rapa 20–23	Pool	M	153	854	11	0.024	1100	5	0.08
Controls	Pool	F	280	905			1095		
Rapa 20 mon	Pool	F	136	1040	15	<0.0001	1231	12	<0.0001
Rapa cycles	Pool	F	136	977	8	<0.0001	1203	10	0.0004
Rapa 20–23	Pool	F	136	940	4	0.15	1136	4	0.12

Note

*p*‐Values derived from log‐rank test, stratified by site, calculated for each sex separately. The Wang–Allison (WA) test is described in Section 4.

In contrast, each of the three protocols produced a distinctly different level of benefit in female mice. Rapa 20 led to a 15% increase in median (*p* < 0.0001 by log‐rank test), while Rapa cycles led to an 8% increase (*p* < 0.0001) and Rapa 20–23 led to a mere 4% increase in median survival (*p* = 0.15). The first two of these protocols also led to a significant result with the WA test (*p* < 0.0004 in each case), but Rapa 20–23 did not (*p* = 0.12). Pairwise comparisons among the three dose protocols by log‐rank test showed that each one differed from the other two, with *p*‐values ranging from 0.04 to 0.0001.

Although our pre‐specified analytical method gives first priority to evaluation of the pooled data sets, we also evaluate site‐specific results, even though each site has only one third of the mice in the pooled set, and therefore far lower statistical power. Survival plots for each combination of sex, site, and Rapa protocol are shown in Figure S1, and site‐specific statistics are collected in Table S1. These revealed a striking degree of site‐specific variation. For female mice, each site showed the same pattern, that is, significant effects of the Rapa 20 and Rapa cycles protocol, and the absence of significant benefit using the Rapa 20–23 protocol, but the extent of change in median varied a great deal, with the UM females typically showing the smallest percentage increase in median lifespan. All three sites showed a similar degree of increase in the 90th percentile age for the Rapa 20 and Rapa cycles treatment group. In males, however, responses to Rapa 20 varied from 0% to 16% increase in median (rank order TJL < UM < UT), responses to Rapa cycles varied from −5% to 41% (rank order UT < TJL < UM), and responses to Rapa 20–23 varied from −11% to 13% (TJL < UM = UT).

Inspection of the site‐specific survival plots suggests the possibility of unanticipated artifacts with effects on lifespan because survival curves at several sites appear to show differences between Control and treatment cohorts prior to the initiation of Rapa administration at age 20 months (604 days). At UM, for example, each of the three groups of male mice, randomly assigned at weaning to one of the Rapa treatment protocols, had fewer deaths than the Control mice prior to the onset of Rapa treatment at 604 days. Similar phenomena are visible in Figure S1 for TJL females and UT males. We suspect this may reflect unanticipated differences between the chow provided to these mice prior to 604 days; although both the Control cages and those destined for Rapa treatment received food with the same formula (Purina 5LG6), mice in the Control group received chow prepared, by TestDiets, using the equipment employed for production of all ITP drug/food mixtures, while mice in the Rapa group received 5LG6 from a different supplier prior to 604 days of age.

**FIGURE 2:**
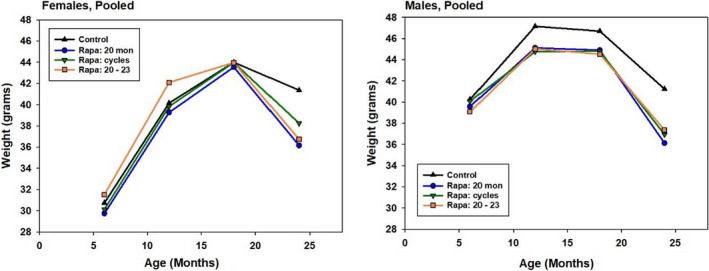
Weight at 6–24 months of age for Control and Rapa‐treated mice, pooled across sites. Each symbol represents the mean of 295 male and 298 female control mice at 6 months, falling to 125 males and 210 females at 24 months. Corresponding values for each Rapa group are an average of 150 males and 135 females at 6 months, falling to an average of 81 males and 102 females at 24 months. SEM values ranged from 0.3 to 0.8 g. All groups of Rapa males differed from Controls at *p* < 0.014 at ages 12, 18, and 24 months (Tukey's post hoc test after ANOVA, stratified by site.) All Rapa females differed from Controls at *p* < 0.001 at 24 months only. The “Rapa 20–23” group of females also differed from Controls (*p* = 0.03) at 12 months

Mice in the longevity cohorts were weighed at 6, 12, 18, and 24 months, and the mean levels for Control and the Rapa groups, averaged across sites, are shown in Figure 2. Control females gained weight until 18 months, but lost weight between 18 and 24 months, in this cohort as in previous ITP cohorts. Each of the three Rapa protocols accelerated the weight loss between Control and Rapa females after 18 months. Females in all three Rapa groups were significantly lighter (*p* < 0.001) than Controls at 24 months, but there were no significant differences in weight at 18 months. In contrast, males in each of the Rapa‐assigned groups were lighter than Control males at 12 and at 18 months of age (*p* < 0.005 in each case), that is, prior to their first exposure to Rapa at 20 months of age, consistent with the idea that unanticipated differences between the two sources of drug‐free 5LG6 mouse chow might have contributed to the divergence of survival curves prior to 20 months of age.

**FIGURE 3:**
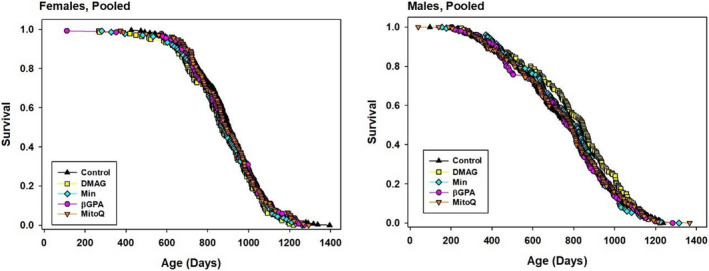
Kaplan–Meier survival plots for female and male mice treated with 17‐DMAG, Min, bGPA, or MitoQ

The cohort initiated in 2015 also included groups of mice given 17‐DMAG (30 ppm, from 6 months), Min (300 ppm, from 6 months), β‐GPA (3300 ppm, from 6 months), or MitoQ (100 ppm, from 7 months). The survival curves are shown in Figure 3, and statistics for these agents are collected in Table 2. None of the drugs led to a significant change in lifespan as indicated by the log‐rank test, and none improved the proportion of mice surviving to the 90th percentile age as tested by the WA test. Corresponding statistics for each individual site are presented in Table S2. None of these four agents produced a significant change in lifespan at any site.

**TABLE 2:** Survival statistics for mice treated with 17‐DMAG, Min, bGPA or MitoQ, pooled across sites.

Rx	Group	Sex	Count	Median (days)	Percent change in median	Log‐rank *p*‐value	90th %ile (days)	Percent change in 90th %ile	WA *p*‐value
Controls	Pool	M	300	772			1049		
17‐DMAG	Pool	M	156	844	9	0.12	1090	4	0.13
Min	Pool	M	156	807	5	0.99	1026	−2	0.50
bGPA	Pool	M	156	764	−1	0.73	1054	0	0.86
MitoQ	Pool	M	156	784	2	0.91	1066	2	0.86
Controls	Pool	F	280	905			1095		
17‐DMAG	Pool	F	136	866	−4	0.12	1074	−2	0.29
Min	Pool	F	136	878	−3	0.38	1094	0	0.99
bGPA	Pool	F	136	893	−1	0.91	1114	2	0.39
MitoQ	Pool	F	136	890	−2	0.67	1105	1	0.39

Note

*p*‐Values derived from log‐rank test, stratified by site, calculated for each sex separately. The Wang–Allison (WA) test is described in Section 4.

## 3 DISCUSSION

Previous ITP papers have reported that encapsulated rapamycin (eRapa) extends lifespan (including an index of maximal lifespan) when given at 9 months of age at doses of 4.7, 14, and 42 ppm in food, with similar effects when given at 14.7 ppm (equivalent to 2 mg/kg body weight per day) starting at 20 months (Harrison et al., 2009; Miller et al., 2011, 2014). A dose in food of 42 ppm is equivalent to a dose of 7 mg/kg body weight per day for a 30 g mouse eating 5 g of chow per day. Other groups (Anisimov et al., 2011; Fok et al., 2013) have also documented lifespan extension in C57/BL6 and 129/Sv mice, over doses ranging from 0.7 mg/kg body weight to 8 mg/kg body weight. However, negative side effects have been noted in mice treated with rapamycin, including cataracts, testicular degeneration (Wilkinson et al., 2012), insulin resistance (Fok et al., 2013), and glucose intolerance (Lamming et al., 2012; Miller et al., 2014). One approach to reducing unwanted side effects of therapeutic agents is intermittent dosing, a common practice in cancer chemotherapy. Intermittent dosing has been reported to decrease rapamycin‐induced glucose intolerance, reduce rapamycin‐induced loss of testicular weight and lessen the effects of rapamycin on the immune system in 9‐month‐old male C57BL/6 mice (Arriola Apelo, Neuman, et al., 2016).

In this study, we compared three different rapamycin dosing regimens, in parallel, for their effects on longevity. Intermittent treatment with rapamycin (2 mg/kg body weight per day, i.p.) administered once every 5 days starting at 20 month of age reportedly extended lifespan in female C57BL/6 mice (Arriola Apelo, Pumper, et al., 2016). Moreover, intermittent treatment of female 129/Sv mice with rapamycin 1.5 mg/kg body weight, three times a week for a period of 2 weeks, followed by 2 weeks without rapamycin, also increased lifespan when started at 2 months of age (Anisimov et al., 2011). Chen et al. (2009) reported that rapamycin administered at 4 mg/kg body weight by intraperitoneal injection to 22‐ to 24‐month‐old C57BL/6 male mice, every other day for only 6 weeks, increased longevity (death ratio of 0.8 versus 0.2 death ratio at 30 weeks after first treatment). In the present study, we compared dosing schemes for microencapsulated rapamycin (eRapa) for their effects on survival, comparing continuous administration from 20 months, 3‐month exposure from 20 to 23 months, and treatment every other month beginning at 20 months of age. Continuous treatment with 42‐ppm eRapa starting at 20 months of age increased median and 90th percentile lifespan in both males and females. Treatment with eRapa every other month starting at 20 months of age increased median and 90th percentile lifespan in both males and females. Treatment with eRapa, starting at 20 months and stopped at 23 months of age, increased median lifespan in males only but did not lead to a significant effect on our measure of survival to the 90th percentile lifespan in either sex.

In a previous paper, in which Rapa was used at 42 ppm starting at age 9 months (Miller et al., 2014), median survival was increased by 23% in males and 26% in females. Each value is substantially higher than the corresponding changes seen in the current paper, (i.e., 11% in males and 15% in females), when the same dose of 42 ppm was initiated at 20 months of age. Although it is always hazardous to compare results from studies done many years apart, that is, using “historical” data rather than contemporaneous data sets, it may be that this Rapa dose is more effective when given starting earlier in adult life. This is in contrast to our evidence that the 14 ppm dose is equally effective regardless of age (Harrison et al., 2009; Miller et al., 2014).

Bitto et al. (2016) evaluated the effects of rapamycin given for a 3‐month period starting at 20–21 months of age, using C57BL/6 mice and a dose, in chow, of 126 ppm, that is, 3‐fold higher than the dose we used in the current study. Treated mice had a median survival of approximately 996 days, a 12.5% increase over the control value of 885 days, pooled across sex. The paper did not include a formal test of maximum lifespan, but inspection of their Figure 4 suggests that their 3‐month regime, equivalent to our Rapa 20–23 protocol, may have had more late‐life benefit in male C57BL/6 mice than in females. Although differences in base diet, mouse stock, and vivarium conditions make a direct comparison to our own study impossible, it is of interest that the 12.5% increase survival in this 3‐month transient protocol is similar to that which we see in our Rapa 20–23 males (11%) given the 42 ppm dose, though higher than the 4% increase seen in our female mice.

Our previous study (Miller et al., 2014) of the effects of different doses of Rapa on lifespan showed that the increased survival in response to increasing concentrations of rapamycin was associated with higher blood levels of Rapa in females. We do not have a ready explanation for the sexual dimorphism we have noted in responses to the Rapa 20–23 dosing regime in the present study and would need to conduct detailed pharmacodynamic analysis of Rapa fluctuations at several intervals after initiation of these dosing strategies. It is possible, in addition, that some of the Rapa effects involve alteration in microbial populations within the GI tract, which could show variable time courses of drug‐induced reconfiguration and recovery. Developing clinical strategies for optimal benefit in long‐term Rapa treatment, with minimal side effects may require careful stepwise refinement and adjustment for sex‐specific effects.

Four other agents were tested in the C2015 cohort, but none of these led to any improvement in lifespan. Each of the four had a plausible rationale for inclusion the study:

17‐dimethylaminoethylamino‐17‐demethoxygeldanamycin hydrochloride (17‐DMAG) is a third generation, orally bioavailable, specific HSP90 inhibitor (Jez et al., 2003). 17‐DMAG is a chemically modified version of Geldanamycin, a benzoquinone antibiotic initially discovered in the organism *Streptomyces hygroscopicus*. 17‐DMAG effectively inhibits HSP90, has diverse anti‐tumor actions (Ikebe et al., 2013), and suppresses neurotoxicity in lower organisms such as *D. melanogaster* (Zhang et al., 2014). In addition, it has a neuro‐protective role in mice (Silva‐Fernandes et al., 2014), improves survival of animals exposed to near‐lethal doses of radiation (Lu et al., 2013), and reduces inflammation (Madrigal‐Matute et al., 2010, 2012). This spectrum of results suggested the idea that lifelong exposure to 17‐DMAG might increase mouse longevity, but our data give no support for this hypothesis. The concentrations of 17‐DMAG in the food were verified in six different batches of food sampled over a 3 to 4‐year period (see Methods S1 for methods of analysis). The mean ± SEM of the concentrations of 17‐DMAG in the chow was 30.00 ± 2.18, which matches the intended concentration. Thus, differences in concentration in the chow do not explain the current findings. It is possible that a different dose of 17‐DMAG may have a positive effect on longevity.

MitoQ is a small molecule antioxidant that accumulates in mitochondria in cultured cells and in vivo (Kelso et al., 2001; Smith et al., 2003; Smith et al., 2010). Orally administered MitoQ reportedly decreases pathology when applied to rodent models of age‐related human diseases involving mitochondrial oxidative damage (Smith et al., 2011; Smith et al., 2010). These include models of cardiac ischemia‐reperfusion injury (Adlam et al., 2005), Alzheimer's disease (McManus et al., 2011), Parkinson's disease (Ghosh et al., 2010), amyotrophic lateral sclerosis (Miquel et al., 2014), diabetic nephropathy (Chacko et al., 2010), age‐related arterial endothelial dysfunction (Gioscia‐Ryan et al., 2014), and age‐related macular degeneration (Tarallo et al., 2012). MitoQ has also been administered to humans in three studies (Gane et al., 2010; Rossman et al., 2018; Snow et al., 2010) and was able to reverse age‐associated vascular dysfunction in old but healthy individuals (Rossman et al., 2018). Despite this variety of positive effects on health indices in these various disease models, there were no effects on longevity in the present study. The concentration of MitoQ in the food was verified in four different batches of food sampled over a 3‐year period (see Methods S2 for methods of analysis). The mean ± SEM of the concentrations of MitoQ in the chow was 88.00 ± 19.95 ppm which is consistent with the intended concentrations of MitoQ in the food. Hence, a lack of effect of MitoQ on lifespan is not due to instability of the drug in the food. In another study, however, the levels of MitoQ in the hearts and livers in the treated mice were below the detection limits when measured by LC‐MS/MS relative to deuterated internal standards (Rodriguez‐Cuenca et al., 2010). Hence, tissues levels in the mice in this study were at least ~20‐fold lower than the levels that showed efficacy in previous mouse models of diseases. The low levels of MitoQ may be due to the poor bioavailability of MitoQ when mixed with solid food due to its strong tendency to adsorb to hydrophobic surfaces (Smith & Murphy, 2010). Therefore, it is not possible to make any robust conclusions on the impact of MitoQ on lifespan from this study and further studies at doses higher than the one chosen in the present study may produce a different outcome.

Minocycline belongs to a class of semi‐synthetic antibiotics that includes tetracycline, doxycycline and demeclocycline. Minocycline has been found to extend lifespan in worms and Drosophila (Oxenkrug et al., 2012; Ye et al., 2014) and to have beneficial effects in a wide range of rodent models of human diseases. Minocycline leads to improved outcomes in a mouse model of atherosclerosis (Shahzad et al., 2011), the transgenic APP/presenilin mouse model of Alzheimer's disease (Biscaro et al., 2012), the MPTP mouse model of Parkinson disease (Du et al., 2001) and after whole‐body γ‐irradiation in mice (Mehrotra et al., 2013) to name a few. Minocycline has also been reported to have benefits in human trials. It was reported to improve refractory rheumatoid arthritis, to improve recovery in patients with acute stroke, to improve symptoms of fragile X syndrome, and to improve negative symptoms in early schizophrenia (Chaudhry et al., 2012; Lampl et al., 2007; Langevitz et al., 1992; Miyaoka et al., 2012; O'Dell et al., 1997; Paribello et al., 2010; Smith et al., 2011). However, 300 ppm minocycline did not modify lifespan in UM‐HET3 mice. The concentration of minocycline in the food was verified in 6 different batches of food sampled over a four‐year period (see Methods S3 for methods of analysis). The mean (SEM) concentration of βGPA in six batches of food pellets was 308 ± 23.2 (SEM) ppm, which was 102% of the expected concentration of 300 ppm on average. However, it is possible that concentrations higher or lower than 300 ppm may produce beneficial effects on longevity.

β‐GPA is a creatine analog that stimulates AMP‐activated protein kinase by depleting cellular energy stores (Bergeron et al., 2001). β‐GPA treatment produces beneficial physiological effects that are associated with some (but not all) longevity extending treatments (Bergeron et al., 2001; Neubauer et al., 1998; Zong et al., 2002). For example, β‐GPA treatment of mice significantly lowers both blood glucose and blood insulin levels, similar to the effects of dietary restriction (Ohira et al., 1994; Reznick et al., 2007). Glucose tolerance is also improved in β‐GPA treated animals, though these effects are greater in diabetic animals compared with control animals (Meglasson et al., 1993). In the present study, β‐GPA was added to food at a concentration of 3300 ppm and fed to mice beginning at 6 months of age. The concentration of β‐GPA in the food was verified in six different batches of food sampled over a 4‐year period 3248 ± 116 (SEM) ppm (see Methods S4 for methods of analysis). Despite the beneficial physiological effects reported in various animal models, we observed no effects of β‐GPA on survival in the present study. This is consistent with the results of a recent study in which β‐GPA treatment had no effect on increasing survival in three different strains of *C. elegans* (Coleman‐Hulbert et al., 2020). In that study, an effect on survival was detected in only one strain, and in that strain, β‐GPA reduced lifespan.

## 4 EXPERIMENTAL PROCEDURES

### 4.1 Animals

UM‐HET3 mice were produced at each of the three test sites as previously described in detail (Harrison *et al*. 2014; Miller et al., 2011; Strong et al., 2016, 2013). The mothers of the test mice were CByB6F1/J, JAX stock #100009, whose female parents are BALB/cByJ and whose male parents are C57BL/6J. The fathers of the test mice were C3D2F1/J, JAX stock #100004, whose mothers are C3H/HeJ and whose fathers are DBA/2J. For breeding cages, each site used Purina 5008 mouse chow. For weanlings prior to 4 months of age, each site used Purina 5LG6.

Mice were housed as previously described (Strong et al., 2013) in plastic cages with metal tops, using 1/4 inch corn‐cob bedding (Bed O'Cobs, produced by The Andersons). Mice were given free access to water, acidified (pH 2.5–2.7) by addition of hydrochloric acid, using water bottles rather than an automated watering system. Mice were housed in ventilated cages and were transferred to fresh cages every 14 days. Temperature was maintained within the range of 21°C to 23°C.

At the age of 42 days, each cage was assigned to a control or test group by use of a random number table. Each mouse was then briefly anesthetized by isoflurane inhalation administered either by nose cone or by an instrument designed for small animal anesthesia and a radio‐frequency identification chip was implanted by sterile syringe beneath the dorsal skin between the shoulder blades, after which the wound was closed by a drop of superglue (Loctite gel, purchased locally, or Nexaband S/C, purchased from Abbott Laboratories). UM and UT used chips purchased from AVID Microchip ID Systems (Catalog AVID3002); TJL used chips purchased from Locus Technology (catalog 1D‐100A). A portion of the distal tail (1 cm) was taken and frozen for later analysis of DNA polymorphisms, after which the mouse was permitted to awaken from the anesthesia. The duration of anesthesia was approximately 1–2 min.

Details of the methods used for health monitoring were provided in Miller et al. (2014); in brief, each of the three colonies was evaluated four times each year for infectious agents, including pinworm. All such tests were negative throughout the entire study period.

### 4.2 Control and experimental diets

Mice were originally weaned onto 5LG6 from Purina. All diets containing drugs were prepared by TestDiet, Inc., a division of Purina Mills. These diets were all based on the Purina 5LG6 mouse chow. Test Diets also provided a Control chow processed in the same way but with no added drugs, which we refer to as “Test Diets control chow.” “Test Diets control chow” and mouse chow containing each of the test substances were prepared at intervals of approximately 4 months. Mice in the Control group received Test Diets Control Chow (5LG6 formulation), without drugs added, from 6 months of age. Mice in cages destined to receive Rapa were given 5LG6 from Purina (not Test Diets Control Chow) until Rapa was started at 20 months. Each batch of food was shipped at the same time to each of the three test sites. 17‐DMAG was obtained from Selleckchem Chemicals LLC. It was mixed with chow at a concentration of 30 ppm and fed to mice beginning at 6 months of age. Mito Q was a gift from Mr. Greg McPherson, CEO of MitoQ Inc (Auckland, NZ). It was mixed with chow at a concentration of 100 ppm and fed to mice beginning at 7 months of age. Minocycline was obtained from the UM pharmacy. It is distributed by Actavis Pharma, Inc. It was mixed with chow at a concentration of 300 mg/kg of food (300 ppm) and fed to mice beginning at 6 months of age. β‐GPA was purchased from Pure Chemistry Scientific Inc., mixed with chow at a concentration of 3300 ppm, and fed to mice beginning at 6 months of age. Microencapsulated rapamycin was obtained from Emtora Biosciences (formerly Rapamycin Holdings) and mixed at a concentration of 42 ppm. This mixture was fed to mice starting at 20 months of age.

### 4.3 Removal of mice from the longevity population

As described in detail in Miller et al. (2011), mice were removed from the study because of fighting, or accidental death, typically during chip implantation, or because of chip failure, or because they were used for another experimental purpose, such as testing for blood levels of a test agent. If used for experimental purposes, the mice were removed randomly from the colony. For survival analyses, all such mice were treated as alive at the date of their removal from the protocol and lost to follow‐up thereafter. These mice were not included in calculations of median longevity.

### 4.4 Estimation of age at death (lifespan)

Mice were examined twice daily for signs of ill health. Mice were euthanized for humane reasons if so severely moribund that they were considered, by an experienced technician, unlikely to survive for more than an additional 48 h. A mouse was considered severely moribund if it exhibited more than one of the following clinical signs: (a) inability to eat or to drink; (b) severe lethargy, as indicated by reluctance to move when gently prodded with a forceps; (c) severe balance or gait disturbance; (d) rapid weight loss over a period of 1 week or more; or (e) an ulcerated or bleeding tumor. The age at which a moribund mouse was euthanized was taken as the best available estimate of its natural lifespan. Mice found dead were also noted at each daily inspection.

### 4.5 Statistical methods

For each sex, we performed site‐specific and combined site analysis. We calculated the median survival for the control group as well as for each treatment group. To compute the median percentage increase, we subtracted the median age in the control group from the corresponding value in the treatment group and divided the difference by median age of the control group and multiplied by 100. Using a two‐sided 5% significance level, we performed the log‐rank test to determine whether survival curves for mice receiving treatment differ from the survival function for control mice. Log‐rank tests that pooled data across the three test sites used a method that stratifies by site. To assess the maximum lifespan, we computed 90th percentile age of both the treated and control mice. To determine which treatments prolonged longevity in mice, we utilized the Wang–Allison test (Wang et al., 2004). This is the Fisher exact test comparing the numbers of mice surviving in control and treatment group at the age corresponding to the 90th percentile of lifespan in the joint survival distribution. We further assessed the longevity of mice in all three sites combined using a modified version of the Wang–Allison test in the manner with which the 2 × 2 contingency table is constructed separately for each site. Basically, we report the sum of corresponding site‐specific 2 × 2 tables cell entries as the combined site 2 × 2 table cell entries. This allows for information from all sites to be used in a balanced manner.

## CONFLICT OF INTEREST

The University of Texas Health Science Center at San Antonio has applied for a patent, U.S. Patent Application No. 13/128,800, by inventors Zelton Dave Sharp and Randy Strong, for an encapsulated rapamycin formulation used in this paper. Under a licensing agreement between Emtora Biosciences (formerly Rapamycin Holdings, Inc.) and the University of Texas Health Science Center San Antonio, R. Strong, and Z.D. Sharp, the University is entitled to milestone payments and royalty on sales of microencapsulated rapamycin. The university has a plan for managing conflicts of interest under its “Policy and Procedures for Promoting Objectivity in Research by Managing, Reducing or Eliminating Conflicts of Interest.” Michael P. Murphy consults for Antipodean Pharmaceuticals Inc., which is developing MitoQ as a potential therapy and also holds patents in the use of MitoQ.

## AUTHOR CONTRIBUTIONS

D.E.H., R.S., and R.A.M. are the principal investigators at the three collaborating institutions and are responsible for project design, supervision of technical personnel, interpretation of results, and preparation of manuscript drafts. J.F.N. and P.R. provided advice on experimental design and interpretation, and comments on the manuscript. E.F supervised laboratory personnel and data collection at UT. M.A.J. supervised assays at the UT site. F.M. served as the project officer for the National Institute on Aging and contributed to program development, experimental design, and analysis. A.R. proposed intermittent rapamycin for study; M.P.M. proposed MitoQ for study; N.M. and A.B.S. proposed β‐GPA for study; M.P.M. proposed minocycline for study; S.L. suggested the 17‐DMAG study.

## Supporting information

Fig S1

Supplementary Material

## Data Availability

The data that support the findings of this study are openly available in The Jackson Laboratory Mouse Phenome Database.at https://phenome.jax.org/projects/ITP1 once the paper is published.
