# Supplementary material for: Rapamycin‐mediated mouse lifespan extension: Late‐life dosage regimes with sex‐specific effects
Source: Aging Cell. 2020 Nov 4;19(11):e13269. doi: 10.1111/acel.13269 (PMC7681050; doi:10.1111/acel.13269)
Supplement: Supplementary file 1 — Fig S1 [file ACEL-19-e13269-s001.docx]

Supplemental Figure 1: Kaplan-Meier survival plots for female and male mice exposed to different Rapa treatment schedules. Green circles: Rapa given from 22 months until death. Red squares: Rapa given from 20-21, 22-23, 24-25, etc., until death. Cyan diamonds: Rapa given for three months only, starting at 20 months. Data are shown separately for each test site.

| 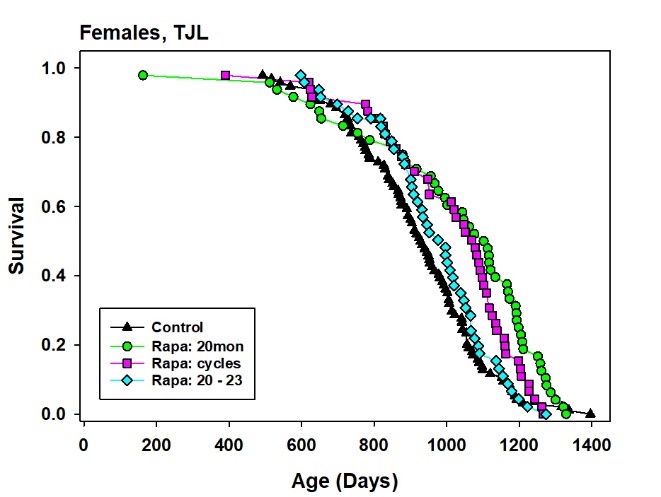 | 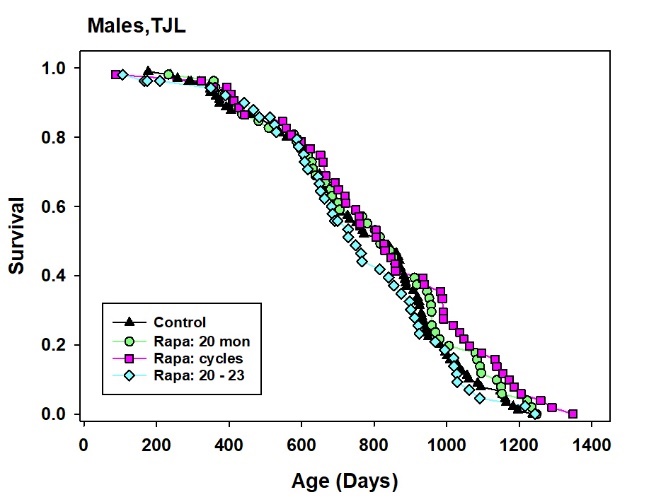 |
| --- | --- |
| 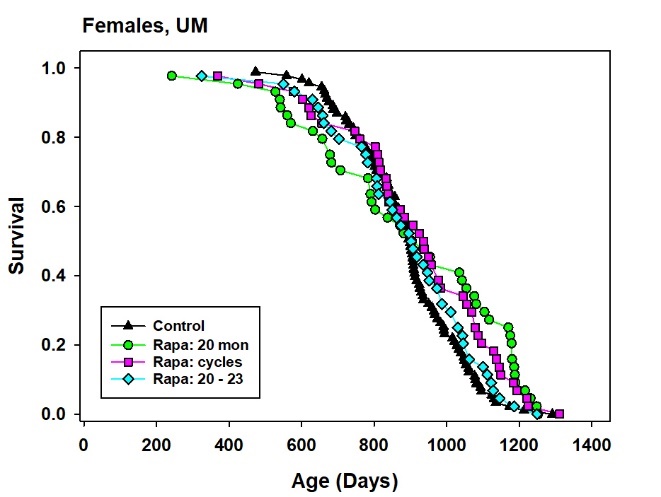 | 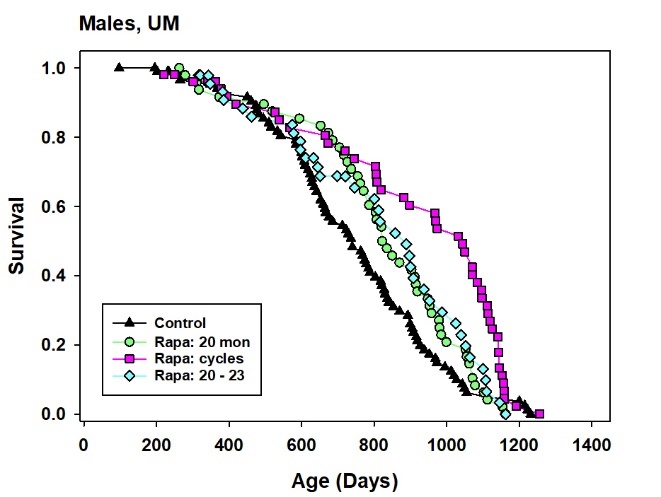 |
| 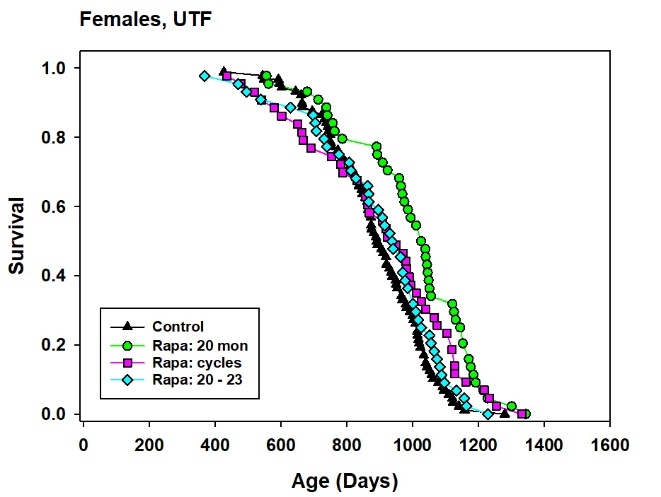 | 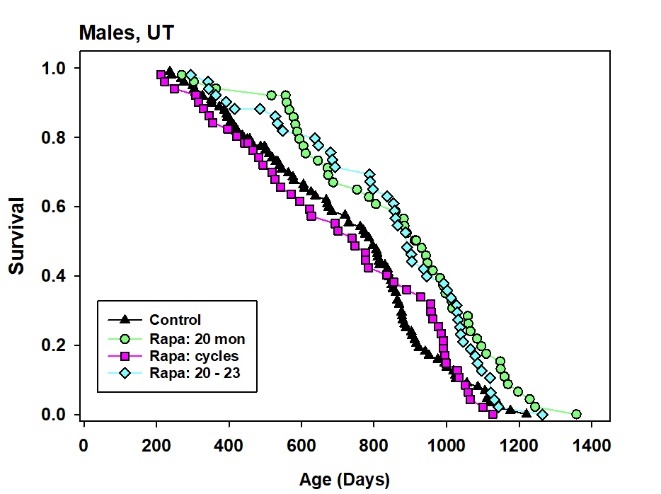 |
