# Supplementary material for: Rapamycin‐mediated mouse lifespan extension: Late‐life dosage regimes with sex‐specific effects
Source: Aging Cell. 2020 Nov 4;19(11):e13269. doi: 10.1111/acel.13269 (PMC7681050; doi:10.1111/acel.13269)
Supplement: Supplementary file 2 — Supplementary Material [file ACEL-19-e13269-s002.docx]

**Supplemental Table S1: Survival statistics for mice treated with Rapamycin using various dosage schedules, shown separately for each site.** P-values derived from log-rank test, calculated for each sex separately. The Wang/Allison (WA) test is described in the Methods.

| **Rx** | Group | Sex | **Count** | **Median (Days)** | **Percent Change in Median** | **Log-rank  p-value** | **90**­th  **%ile (Days)** | **Percent Change in 90th %ile** | **WA p-value** |
| --- | --- | --- | --- | --- | --- | --- | --- | --- | --- |
| Controls | TJL | M | 102 | 819 |  |  | 1061 |  |  |
| Rapa 20 mon | TJL | M | 54 | 817 | 0 | 0.36 | 1138 | 7 | 0.40 |
| Rapa cycles | TJL | M | 54 | 826 | 1 | 0.07 | 1173 | 11 | 0.16 |
| Rapa 20 - 23 | TJL | M | 54 | 729 | -11 | 0.80 | 1029 | -3 | 0.99 |
| Controls | UM | M | 99 | 740 |  |  | 1027 |  |  |
| Rapa 20 mon | UM | M | 51 | 829 | 12 | 0.12 | 1078 | 5 | 0.07 |
| Rapa cycles | UM | M | 54 | 1044 | 41 | 0.0002 | 1156 | 13 | 0.006 |
| Rapa 20 - 23 | UM | M | 48 | 837 | 13 | 0.14 | 1107 | 8 | 0.18 |
| Controls | UT | M | 99 | 786 |  |  | 1025 |  |  |
| Rapa 20 mon | UT | M | 51 | 912 | 16 | 0.0006 | 1169 | 14 | 0.07 |
| Rapa cycles | UT | M | 51 | 744 | -5 | 0.93 | 1052 | 3 | 0.99 |
| Rapa 20 - 23 | UT | M | 51 | 891 | 13 | 0.009 | 1122 | 9 | 0.24 |
|  |  |  |  |  |  |  |  |  |  |
| Controls | TJL | F | 96 | 928 |  |  | 1156 |  |  |
| Rapa 20 mon | TJL | F | 48 | 1108 | 19 | 0.0007 | 1275 | 10 | 0.001 |
| Rapa cycles | TJL | F | 48 | 1074 | 16 | 0.009 | 1226 | 6 | 0.08 |
| Rapa 20 - 23 | TJL | F | 48 | 986 | 6 | 0.42 | 1169 | 1 | 0.99 |
| Controls | UM | F | 92 | 899 |  |  | 1079 |  |  |
| Rapa 20 mon | UM | F | 44 | 920 | 2 | 0.02 | 1189 | 10 | 0.0003 |
| Rapa cycles | UM | F | 44 | 937 | 4 | 0.03 | 1184 | 10 | 0.01 |
| Rapa 20 - 23 | UM | F | 44 | 905 | 1 | 0.48 | 1123 | 4 | 0.23 |
| Controls | UT | F | 92 | 892 |  |  | 1078 |  |  |
| Rapa 20 mon | UT | F | 44 | 1033 | 16 | <0.0001 | 1192 | 11 | < 0.0001 |
| Rapa cycles | UT | F | 44 | 949 | 6 | 0.04 | 1162 | 8 | 0.006 |
| Rapa 20 - 23 | UT | F | 44 | 940 | 5 | 0.32 | 1097 | 2 | 0.55 |

**Supplemental Table S2: Survival statistics for mice treated with 17-DMAG, Min, bGPA, or MitoQ, shown separately for each site.** P-values derived from log-rank test, calculated for each sex separately. The Wang/Allison (WA) test is described in the Methods.

| **Rx** | Group | Sex | **Count** | **Median (Days)** | **Percent Change in Median** | **Log-rank  p-value** | **90**­th  **%ile (Days)** | **Percent Change in 90th %ile** | **WA p-value** |
| --- | --- | --- | --- | --- | --- | --- | --- | --- | --- |
| Controls | TJL | M | 102 | 819 |  |  | 1061 |  |  |
| 17-DMAG | TJL | M | 54 | 833 | 2 | 0.41 | 1102 | 4 | 0.41 |
| Min | TJL | M | 54 | 809 | -1 | 0.30 | 1028 | -3 | 0.57 |
| bGPA | TJL | M | 54 | 766 | -6 | 0.67 | 1057 | 0 | 0.99 |
| MitoQ | TJL | M | 54 | 800 | -2 | 0.60 | 1116 | 5 | 0.41 |
| Controls | UM | M | 99 | 740 |  |  | 1027 |  |  |
| 17-DMAG | UM | M | 51 | 841 | 14 | 0.92 | 1005 | -2 | 0.37 |
| Min | UM | M | 51 | 844 | 14 | 0.44 | 1027 | 0 | 0.99 |
| bGPA | UM | M | 51 | 749 | 1 | 0.64 | 1046 | 2 | 0.76 |
| MitoQ | UM | M | 51 | 769 | 3 | 0.37 | 1021 | -1 | 0.77 |
| Controls | UT | M | 99 | 786 |  |  | 1025 |  |  |
| 17-DMAG | UT | M | 51 | 856 | 9 | 0.08 | 1106 | 8 | 0.40 |
| Min | UT | M | 51 | 769 | -2 | 0.76 | 987 | -4 | 0.79 |
| bGPA | UT | M | 51 | 818 | 4 | 0.77 | 1025 | 0 | 0.99 |
| MitoQ | UT | M | 51 | 784 | 0 | 0.63 | 1066 | 4 | 0.99 |
| Controls | TJL | F | 96 | 928 |  |  | 1156 |  |  |
| 17-DMAG | TJL | F | 48 | 903 | -3 | 0.31 | 1091 | -6 | 0.55 |
| Min | TJL | F | 48 | 922 | -1 | 0.77 | 1116 | -3 | 0.77 |
| bGPA | TJL | F | 48 | 954 | 3 | 0.44 | 1086 | -6 | 0.39 |
| MitoQ | TJL | F | 48 | 968 | 4 | 0.44 | 1205 | 4 | 0.26 |
| Controls | UM | F | 92 | 899 |  |  | 1079 |  |  |
| 17-DMAG | UM | F | 44 | 854 | -5 | 0.07 | 1002 | -7 | 0.22 |
| Min | UM | F | 44 | 825 | -8 | 0.12 | 1050 | -3 | 0.55 |
| bGPA | UM | F | 44 | 868 | -4 | 0.92 | 1081 | 0 | 0.77 |
| MitoQ | UM | F | 44 | 884 | -2 | 0.56 | 1035 | -4 | 0.38 |
| Controls | UT | F | 92 | 892 |  |  | 1078 |  |  |
| 17-DMAG | UT | F | 44 | 867 | -3 | 0.92 | 1085 | 1 | 0.55 |
| Min | UT | F | 44 | 887 | -1 | 0.78 | 1100 | 2 | 0.77 |
| bGPA | UT | F | 44 | 868 | -3 | 0.50 | 1120 | 4 | 0.07 |
| MitoQ | UT | F | 44 | 870 | -3 | 0.31 | 1068 | -1 | 0.99 |

**Supplemental Methods:**

**Measurement of 17-Dimethylaminoethylamino-17-demethoxygeldanamycin (17-DMAG) Using HPLC-tandem MS.**

All reagents were purchased from Sigma Chemical Company (St. Louis, MO). Milli-Q water was used for preparation of all solutions.

The HPLC system consisted of a Shimadzu SIL 20A HT autosampler, LC-20AD pumps (2), and an AB Sciex API 3200 tandem mass spectrometer with turbo ion spray. The LC analytical column was a C18 Excel 3 ACE PFP (3 x 75 mm, 3 micron) purchased from MacMod (Chadds Ford, PA) and was maintained at 25^o^C during the chromatographic runs using a Shimadzu CT-20A column oven. Mobile phase A contained 99.9% H2O with 0.1% formic acid. Mobile phase B contained 99.9% methanol with 0.1% formic acid. The flow rate of the mobile phase was 0.2 ml/min. 17-DMAG was eluted isocratically with 30% mobile phase A and 70% mobile phase B. The 17-DMAG transition was detected in positive mode at 617.2 Da (precursor ion) and the daughter ion was detected at 524.3 Da. The internal standard (Rapamycin) transition was detected at 931.6 Da (precursor ion) and the daughter ion was detected at 864.5 Da.

17-DMAG and Rapamycin super stock solutions were prepared in methanol at a concentration of 1 mg/ml and stored in aliquots at -80^o^C. Working stock solution were prepared each day from the super stock solutions at concentrations of 100 and 10 μg/ml and used to spike the calibrators.

Measurement of 17-DMAG in Food Pellets-Food calibrator samples were prepared by spiking food samples at concentrations of 0, 5, 10, 20, 50 and 100 ng/mg. 17-DMAG was quantified in mouse food by mixing 20 mg of calibrator and unknown samples with 2 mL of a solution containing 70% methanol and 0.1% formic acid. The samples were then vortexed vigorously and shaken for 20 minutes. Then 300 µL of the solution were transferred to microfilterfuge tubes and centrifuged at 13,000 *g* for 1 minute. The final extracts were transferred to autosampler vials and 10 µL were injected into the LC/MS/MS. The peak area responses of 17-DMAG for each unknown sample were compared against a linear regression of the peak area responses obtained by the calibration samples to quantify 17-DMAG. The concentration of 17-DMAG was expressed as ng/mg food.

**Measurement of MitoQ Using HPLC-tandem MS**

Mitoquinol Mesylate, Mitoquinone Mesylate, and Mitoquinol D15 were provided by Irix Pharmaceuticals (Florence, SC). Milli-Q water was used for preparation of all solutions.

The HPLC system consisted of a Shimadzu SIL 20A HT autosampler, LC-20AD pumps (2), and an AB Sciex API 4000 Qtrap tandem mass spectrometer with turbo ion spray. The LC analytical column was a C18 Excel 3 ACE (3 x 75 mm, 3 micron) purchased from MacMod (Chadds Ford, PA) and was maintained at 25^o^C during the chromatographic runs using a Shimadzu CT-20A column oven. Mobile phase A consisted of 99.9% H2O with 0.1% formic acid. Mobile phase B consisted of 99.9% acetonitrile with 0.1% formic acid. The flow rate of the mobile phase was 0.5 ml/min. Mitoquinol/Mitoquinol were eluted with a gradient. The initial mobile phase was 5% B and at 1 minute after injection was ramped to 99% B. From 4.0 min to 7.0 min the mobile phase was maintained at 99% B and at 7.1 minutes was switched immediately back to 5% B and ran for 2.9 minutes to equilibrate the column before the next injection.

The Mitoquinol transition was detected in positive mode at 585 Da (precursor ion) and the daughter ion was detected at 289 Da. The Mitoquinone transition was detected at 584 Da (precursor ion) and the daughter ion was detected at 442 Da. The internal standard (Mitoquinone D-15) transition was detected at 599 Da (precursor ion) and the daughter ion was detected at 457.2 Da.

Mitoquinone and Mitoquinol, D-15 super stock solutions were prepared in ethanol at a concentration of 1 mg/ml and stored in aliquots at -80^o^C. Working stock solution were prepared each day from the super stock solutions at concentrations of 100 and 10 μg/ml and used to spike the calibrators.

Mitoquinone and Mitoquinol were quantified in food pellets. Food calibrator samples were prepared by spiking 10 mg of ground food samples at concentrations of 0, 10, 25, 50, 100, 250 and 500 ppm. Mitoquinone, and Mitoquinol were quantified in mouse food by mixing 10 mg of calibrator and unknown samples with 1 mL of a solution containing 99.9% Acetonitrile and .1% Formic Acid. The samples were then vortexed vigorously and shaken for 30 minutes. The calibrators and unknowns were then centrifuged at 3200 *g* for 30 minutes. The final extracts were transferred to autosampler vials and 10 µL were injected into the LC/MS/MS. The peak area response for each unknown sample was compared against a linear regression of calibrator peak area responses to quantify Mitoquinone and Mitoquinol. The concentration of Mitoquinone and Mitoquinol was expressed as ng/mg food.

**Measurement of Minocycline Using HPLC-tandem MS**

Minocycline and Tetracycline and all reagents were purchased from Sigma Chemical Company (St. Louis, MO). Milli-Q water was used for preparation of all solutions.

The HPLC system consisted of a Shimadzu SIL 20A HT autosampler, LC-20AD pumps (2), and an AB Sciex API 3200 tandem mass spectrometer with turbo ion spray. The LC analytical column was a C18 Excel 3 ACE (3 x 75 mm, 3 micron) purchased from MacMod (Chadds Ford, PA) and was maintained at 25^o^C during the chromatographic runs using a Shimadzu CT-20A column oven. Mobile phase A contained 99.9% H2O with 0.1% formic acid. Mobile phase B contained 99.9% acetonitrile with 0.1% formic acid. The flow rate of the mobile phase was 0.4 ml/min. Minocycline was eluted isocratically with 70% mobile phase A and 30% mobile phase B. The Minocycline transition was detected in positive mode at 458.2 Da (precursor ion) and the daughter ion was detected at 283.2 Da. The internal standard (Tetracycline) transition was detected at 445.09 Da (precursor ion) and the daughter ion was detected at 410.2 Da.

Minocycline and Tetracycline super stock solutions were prepared in methanol at a concentration of 1 mg/ml and stored in aliquots at -80^o^C. Working stock solution were prepared each day from the super stock solutions at concentrations of 100 and 10 μg/ml and used to spike the calibrators.

Measurement of Minocycline in Food Pellets. Food calibrator samples were prepared by spiking food samples at concentrations of 0, 180, 400, 800, 1600 and 3000 ng/mg. Minocycline was quantified in mouse food by mixing 25 mg of calibrator and unknown samples with 30 µL of 100 µg/mL Tetracycline and 3 mL of a solution containing 70% mobile phase A and 30% mobile phase B. The samples were then vortexed vigorously and shaken for 20 minutes. Then 300 µL of the solution were transferred to microfilterfuge tubes and centrifuged at 13,000 *g* for 1 minute. The final extracts were transferred to autosampler vials and 10 µL were injected into the LC/MS/MS. The ratios of Minocycline peak areas to Tetracycline peak areas for each unknown sample were compared against a linear regression of the ratios obtained by the calibration samples to quantify Minocycline. The concentration of Minocycline was expressed as ng/mg food.

**Measurement of β-guanadinopropionic acid (β-GPA) Using HPLC-tandem MS**

β-GPA was purchased from Sigma Chemical Company (St. Louis, MO). Milli-Q water was used for preparation of all solutions.

The HPLC system consisted of a Shimadzu SIL 20A HT autosampler, LC-20AD pumps (2), and an AB Sciex API 3200 tandem mass spectrometer with turbo ion spray. The LC analytical column was a C18 Excel 3 ACE PFP (3 x 75 mm, 3 micron) purchased from MacMod (Chadds Ford, PA) and was maintained at 25^o^C during the chromatographic runs using a Shimadzu CT-20A column oven. Mobile phase A contained 99.9% H2O with 0.1% formic acid. Mobile phase B contained 99.9% acetonitrile with 0.1% formic acid. The flow rate of the mobile phase was 0.5 ml/min. β-GPA was eluted isocratically with 50% mobile phase A and 50% mobile phase B. The β-GPA transition was detected in positive mode at 132.016 Da (precursor ion) and the daughter ion was detected at 72.1 Da. The internal standard (Rapamycin) transition was detected at 931.6 Da (precursor ion) and the daughter ion was detected at 864.5 Da.

β-GPA and Rapamycin super stock solutions were prepared in methanol at a concentration of 1 mg/ml and stored in aliquots at -80^o^C. Working stock solution were prepared each day from the super stock solutions at concentrations of 100 and 10 μg/ml and used to spike the calibrators.

Measurement of β-GPA in Food Pellets. Food calibrator samples were prepared by spiking food samples at concentrations of 0, 100, 1500, 3000, 4000 and 5000 ng/mg. β-GPA was quantified in mouse food by mixing 10 mg of calibrator and unknown samples with 5 mL of a solution containing 90% mobile phase A and 10% mobile phase B. The samples were then vortexed vigorously and shaken for 20 minutes. Then 300 µL of the solution were transferred to microfilterfuge tubes and centrifuged at 13,000 *g* for 1 minute. The final extracts were transferred to autosampler vials and 5 µL were injected into the LC/MS/MS. The peak area response for each unknown sample was compared against a linear regression of calibrator peak area responses to quantify β-GPA. The concentration of β-GPA was expressed as ng/mg food.

**Measurement of Rapamycin Using HPLC-tandem MS**

Rapamycin, Ascomycin and all reagents were purchased from Sigma Chemical Company (St. Louis, MO). Milli-Q water was used for preparation of all solutions.

The HPLC system consisted of a Shimadzu SIL 20A HT autosampler, LC-20AD pumps (2), and an AB Sciex API 3200 tandem mass spectrometer with turbo ion spray. The LC analytical column was a Grace Alltima C18 (4.6 x 150 mm, 5 micron) purchased from Alltech (Deerfield, IL) and was maintained at 25^o^C during the chromatographic runs using a Shimadzu CT-20A column oven. Mobile phase A contained 10 mM ammonium formate and 0.1% formic acid dissolved in 100% HPLC grade methanol. Mobil phase B contained 10 mM ammonium formate and 0.1% formic acid dissolved in 90% HPLC grade methanol. The flow rate of the mobile phase was 0.5 ml/min. Rapamycin was eluted with a gradient. The initial mobile phase was 100% B and at 0.10 minutes after injection was ramped to 100% A. From 4.0 min to 5.0 min the mobile phase was maintained at 100% A and at 5.1 minutes was switched immediately back to 100% B and ran for 4.9 minutes to equilibrate the column before the next injection. The Rapamycin transition was detected in positive mode at 931.6 Da (precursor ion) and the daughter ion was detected at 864.5 Da. The internal standard (Ascomycin) transition was detected at 809.6 Da and the daughter ion was detected at 756.6 Da.

Measurement of Rapamycin in Food Pellets. Rapamycin and Ascomycin (internal standard) super stock solutions were prepared in methanol at a concentration of 1 mg/ml and stored in aliquots at -80^o^C. A working stock solution was prepared each day from the super stock solutions at a concentration of 10 μg/ml and used to spike the calibrators. Food calibrator samples were prepared by spiking food samples at concentrations of 0, 100, 500, 1000, 2000 ng/mg. Rapamycin was quantified in mouse food by mixing 20 mg of calibrator and unknown samples with 5 µL of 1 mg/mL Ascomycin and 4 mL of mobile phase A then vortexing vigorously and shaking for 20 minutes. Then 300 µL of the solution were transferred to microfilterfuge tubes and centrifuged at 13,000 *g* for 1 minute. The final extracts were transferred to autosampler vials and 10 µL were injected into the LC/MS/MS. The peak area ratios for each unknown sample was compared against a linear regression of calibrator peak area ratios to quantify Rapamycin. The concentration of Rapamycin was expressed as ng/mg food.
